# Case Report: A wrong turn: malpositioned pacemaker leads reveal undiagnosed PAPVD after emergency implantation

**DOI:** 10.3389/fcvm.2025.1683645

**Published:** 2025-09-30

**Authors:** Yakhimovich Yana, Aripov Marat

**Affiliations:** ^1^ Cardiology Department, UMC Heart Center, Astana, Kazakhstan; ^2^ Head of Interventional Cardiology Department, UMC Heart Center, Astana, Kazakhstan

**Keywords:** pacemaker, malpositioned pacemaker leads, echocardiography, sinus venosus defect, PAPVD

## Abstract

Malposition of pacemaker leads into the left heart is a rare but clinically significant complication that can lead to systemic thromboembolism. We report the case of a 78-year-old woman who underwent emergency pacemaker implantation due to sinus node dysfunction. Post-procedural imaging incidentally revealed lead malposition into the left heart. Further investigation using transthoracic and transesophageal echocardiography, along with computer tomography, identified a sinus venosus defect and partial anomalous pulmonary venous drainage. Moreover, malposition of the pacemaker leads was confirmed. Given the patient's age, frailty, and absence of thromboembolic events or significant symptoms, a conservative approach was chosen, and lifelong anticoagulation with warfarin was initiated. Surgical intervention and lead extraction were deferred due to high procedural risk. The patient remained clinically stable with preserved pacemaker function and no complications during follow-up. This case underscores the importance of imaging in detecting anomalies associated with pacemaker lead malposition. Management should be individualized, balancing procedural risk against the potential for thromboembolism.

## Introduction

Malposition of pacemaker leads into the left ventricle is uncommon. In most cases, inadvertent lead placement into the left heart occurs through congenital defects such as a patent foramen ovale or various types of atrial septal defects. Many patients with malpositioned leads remain asymptomatic (1, 2). However, this anomaly may increase the risk of adverse cardiovascular events such as transient ischemic attacks, strokes, and cardiac perforation leading to tamponade (3). We present a case in which malpositioned pacemaker leads incidentally led to the diagnosis of previously unrecognized partial anomalous pulmonary venous drainage (PAPVD).

## Case report

A 78-year-old woman was admitted to our institution 2 weeks after undergoing emergency cardiac pacemaker implantation for evaluation and management of inadvertently positioned pacing leads in the left side of the heart.

The patient's medical history included single-vessel coronary artery disease without prior myocardial infarction, along with well-controlled hypertension. A cardiac pacemaker was implanted in April 2025 due to sinus node dysfunction. The day after pacemaker implantation, a chest x-ray showed a left-sided pneumothorax, which was managed with thoracocentesis. Transthoracic echocardiography (TTE) revealed malpositioned pacing leads within the left heart chambers. Consequently, the patient was referred to our institution for further diagnosis and treatment. The patient was admitted with complaints of dyspnea and fatigue. On physical examination, heart sounds were normal with no murmurs. Lungs were clear to auscultation bilaterally. There was no pedal edema. Before hospitalization, the patient had been receiving rivaroxaban as anticoagulation therapy.

**Figure 1:**
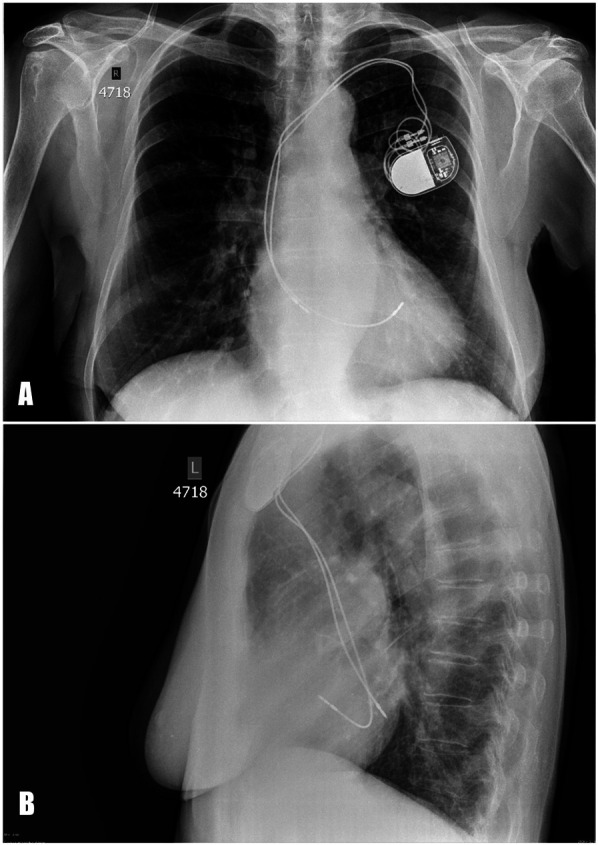
Posteroanterior **(А)** and lateral **(B)** chest x-ray. The pacing leads are abnormally positioned, suggesting placement in the left heart instead of the right.

A blood test revealed an elevated level of NT-proBNP 502 pg/mL. The chest x-ray demonstrated that the pacing lead followed an atypical course (Figure 1). TTE showed abnormal lead positioning, dilatation of right heart chambers, and moderate tricuspid regurgitation.

**Figure 2:**
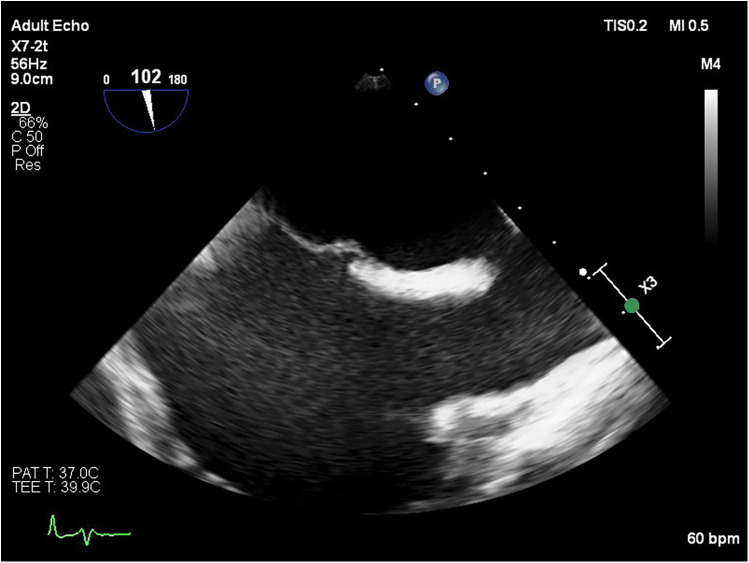
Transesophageal echocardiography image showing a sinus venosus atrial septal defect located near the junction of the SVC and the right atrium.

**Figure 3:**
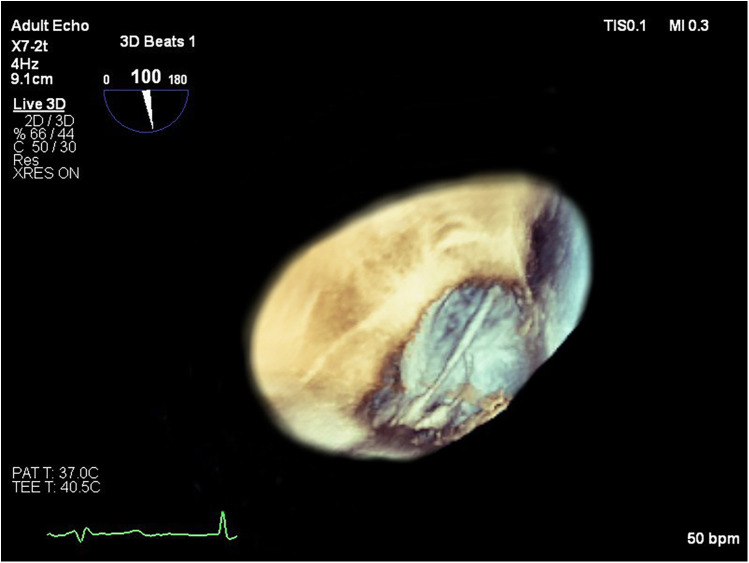
Short-axis left ventricular transesophageal 3D echocardiographic view showing the transit of the two pacing leads across the atrial septal defect. 3D, three-dimensional.

**Figure 4:**
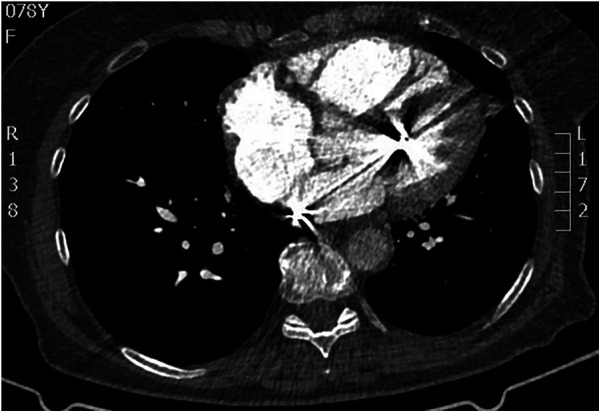
CT scan demonstrating malpositioned cardiac pacing leads located within the left heart.

Transesophageal echocardiography (TEE) demonstrated a superior sinus venosus defect near the junction of the superior vena cava (SVC) and right atrium with left-to-right shunting. Two pacing leads pass through the defect from the SVC into the left side of the heart, with one lead affixed to the left atrium roof and the other extending through the mitral valve without any perforations into the left ventricle, where it is fixed to the interventricular septum. No intracardiac thrombi, or “smoke” and thrombi on pacemaker leads were observed during TEE (Figures 2, 3). A contrast-enhanced computer tomography (CT) scan of the chest was performed to further evaluate the suspected congenital defect and confirm the position of malpositioned pacing leads (Figure 4). CT imaging confirmed the presence of a superior sinus venosus defect located near the junction of the SVC and the right atrium. Moreover, CT revealed partial anomalous pulmonary venous return, with the right superior and middle pulmonary veins draining into the SVC. Given the patient's age, moderate frailty, and absence of significant clinical symptoms, a conservative approach was adopted. Lead extraction was not pursued due to high procedural risk. The patient was managed with lifelong oral anticoagulation with warfarin to mitigate the risk of systemic thromboembolism. Surgical correction of the anomalous pulmonary venous return was not indicated, as the patient remained hemodynamically stable without signs of volume overload. Ongoing follow-up includes regular assessment of anticoagulation status, pacemaker function, and clinical condition. The patient remained clinically stable during follow-up. No thromboembolic or bleeding complications were observed. TTE and x-ray demonstrated stable lead positioning within the left heart chambers, without evidence of thrombus formation or endocardial damage. Pacemaker function remained intact with appropriate sensing and pacing parameters.

## Discussion

Malpositioned leads involving the left heart are associated with a high risk of thrombus formation and systemic thromboembolism, necessitating careful evaluation of therapeutic options (3, 4). The inadvertent placement of leads in the left atrium or ventricle may occur through congenital or acquired interatrial communications. Several cases in the literature have reported pacemaker misplacement into the left ventricle, most often due to previously unrecognized congenital heart defects (5, 6). Management of inadvertent lead malposition in the left heart is controversial due to limited data and depends on timing, symptoms, complications, and comorbidities. Early cases (<3 months) may allow for simple percutaneous removal before fibrous fixation. Chronic cases (>1 year) generally do not require lead removal unless there are strong indications, such as device-related infection (7, 8). Although lead extraction is typically considered to mitigate embolic risk, especially in younger or symptomatic patients, the decision must balance procedural risk against expected benefits. Lead extraction in elderly, frail patients carries a high risk of complications, including cardiac perforation, vascular injury, and death. Anticoagulation therapy plays a pivotal role in the conservative management of patients with left heart lead malposition. Although no large-scale randomized trials exist, several case reports and small series support the use of vitamin K antagonists (VKAs) or direct oral anticoagulation (DOACs) to reduce the risk of systemic embolism (9–11). According to Ohlow et al., long-term anticoagulation with warfarin is generally recommended, as no thromboembolic events were observed in patients who consistently maintained an international normalized ratio (INR) between 2.5 and 3.5 (12).

## Conclusion

The management of patients with malpositioned pacing leads remains non-uniform, due to the lack of standardized guidelines. Therapeutic decisions should be individualized, considering the anatomical anomaly, congenital heart disease, patient comorbidities, and potential complications associated with lead extraction. Further studies are needed to establish evidence-based strategies for optimal management in such cases.

## Data Availability

The raw data supporting the conclusions of this article will be made available by the authors, without undue reservation.
